# Novel all-intra-incisional pin placement technique in robotic total knee arthroplasty: a safer alternative

**DOI:** 10.1186/s42836-025-00329-8

**Published:** 2025-09-02

**Authors:** Zi Qiang Glen Liau, Wai Keong Ryan Loke, Danakkrisna Vachalam S./O. Rangasamie, Yu Liu

**Affiliations:** 1 Alexandra Hospital, Singapore, Singapore; 2 National University Hospital, Singapore, Singapore; 3 Yong Loo Lin School of Medicine, National University of Singapore, Singapore, Singapore

**Keywords:** Robotic, Assisted, Knee replacement, All, Intra, Incisional, Pins, Complications, Pin, Site, Fracture

## Abstract

**Introduction:**

Major robotic systems for total knee replacements necessitate the use of array pins in the tibia and femur. These extra-incisional pins are placed away from the primary incision and may be associated with soft tissue complications and peri-prosthetic fractures. There is currently no standardized, reproducible method for reliably placing pins in the femur and tibia metaphyses. We have developed an all-intra-incisional pin method within the primary incision. This paper aims to describe our technique, analyse the proximity of the pins to the implants, and study complications arising from both techniques.

**Methods:**

A total of 102 robotic-assisted total knee arthroplasties were performed using the ROSA, MAKO, and CORI systems. Patient charts were reviewed for their age, gender, body mass index, and ethnicity. Post-operative day zero radiographs of the operated knee were used for measurements in anteroposterior and lateral views, with X-ray magnifications taken into consideration.

**Results:**

Our study demonstrates that intra-incisional pins can be placed 6.52 times closer to the tibial implant compared to extra-incisional pins on the anteroposterior X-ray view radiographs, with no observed significant difference between the complication rates. In anteroposterior view, it allows placement of tibia pins within 8.99 ± 1.21 mm (95% CI: 7.78, 10.2) of the tibial implant, within 5.93 ± 1.29 mm (95% CI: 4.64, 7.22) of the tibia-reamed-surface, and placement of the femoral pins within 6.01 ± 1.37 mm (95% CI: 4.64, 7.37) of the femoral implant. In the lateral X-ray view, it enables the placement of tibial pins within 9.40 ± 1.43 mm (95% CI: 7.97, 10.8) of the implant. Univariate analysis reveals that our technique and pin-distance from the implants are not influenced by patient demographics.

**Conclusion:**

Our study has demonstrated that our technique is precise, not affected by patients’ demographics, and eliminates the need for pin repositioning, potentially reducing the incidence of pin-site complications.

**Supplementary Information:**

The online version contains supplementary material available at 10.1186/s42836-025-00329-8.

## Introduction

Robot-assisted total knee arthroplasty (TKA) has become increasingly popular globally over the past decade [1]. This rise in adoption has been driven by documented improvements in both clinical and functional outcomes, as well as by enhanced implant positioning precision afforded by robotic technology [2–4]. As part of the surgical workflow, femoral and tibial array pins are placed rigidly into the respective bones [5–7]. These pins allow attachment of the tracker arrays that enable the system to identify the knee’s position accurately within a three-dimensional space, thereby facilitating precise bone cuts and implant placement. According to current technique guides, these pins are typically placed using an extra-incisional approach [5–7]. This involves creating two separate incisions distinct from the primary midline knee incision—one incision over the distal femur and another over the proximal tibia. Although widely practiced, extra-incisional pin placement can potentially lead to several intraoperative and postoperative complications. For example, placing pins in the diaphyseal bone introduces stress risers, weakening the cortex and predisposing patients to pin site and peri-prosthetic fractures [8–10]. Additional morbidity is associated with this technique, including the need for extra stab incisions, increased suture burden, higher infection risk, and the formation of seromas or hematomas. Furthermore, the separate incisions may increase the risk of injuring nearby neurovascular structures, such as the peroneal nerve [8–10]. In fact, the reported rate of pin-site complications was 1.4% in a large cohort study of over 7,000 patients undergoing TKA [11]. Given the steadily increasing number of robotic knee arthroplasties being performed worldwide, evidence-based advancements in pin placement techniques are critical to minimize these complications and optimize patient outcomes [12].

In recent years, some authors have proposed that placing the array pins intra-incisionally—within the main midline incision—may offer a practical solution to mitigate the problems inherent to extra-incisional pin placement [13, 14]. Intra-incisional pinning has the theoretical advantage of reducing the number of incisions required, thereby potentially lowering the risk of wound complications and eliminating the stress risers associated with diaphyseal pin tracks. However, it is important to note that intra-incisional pin placement remains an off-label technique for the majority of commercially available robotic platforms.

While a limited number of studies and case series have described intra-incisional techniques and reported encouraging outcomes [13–15], there remains no anatomically described, universally accepted, reproducible method for consistently placing all pins within the femoral and tibial metaphyses while still ensuring unimpeded surgical access to critical landmarks and bone surfaces throughout each stage of the TKA procedure. Without a validated, standardized approach, there remains uncertainty about whether intra-incisional pin placement can reliably maintain secure array fixation without compromising exposure, implant alignment, or component trialing while reducing associated complications with extra-incisional pin placements. In response to this gap in knowledge, we have developed a novel all-intra-incisional pin placement method designed to achieve secure fixation and minimal complication rates. Our technique enables the pins to remain stable throughout the procedure without obstructing any aspect of the surgical workflow, including bone resections, tibial keel preparation, trial component positioning, or the final placement and assessment of implants. Notably, our approach does not require extending the standard midline incision length, which is an important consideration for maintaining soft tissue integrity and optimizing wound healing.

This paper aims to describe our intra-incisional pinning technique in detail and to analyze the spatial relationship between the pins and the final implant components. By rigorously evaluating the proximity of the pins to definitive implants, we aim to confirm that the pins can be positioned sufficiently close to the implants to avoid collisions during preparation and trialing, yet far enough to maintain a safe working distance for surgical maneuvers. We hypothesize that this technique offers a reproducible, safe, and efficient method to reduce pin-related complications and streamline robotic TKA workflows without compromising surgical exposure or implant positioning accuracy.

## Methods

All methods were carried out in accordance with the Singapore Medical Council’s Ethical Code and Ethical Guidelines (ECEG) 2018 (latest edition). All experimental protocols were approved by the Head of Department of Orthopaedics from both the National University Hospital and Alexandra Hospital. Informed consent was obtained from all the participants themselves as part of their consent-taking process, and none of the participants had legal guardians.

A retrospective study from February 2023 to May 2024 was performed in two tertiary hospitals, using the ROSA (Zimmer), MAKO (Stryker), and CORI (Smith & Nephew) systems. Patients’ electronic medical records (EMR) were reviewed for their age, gender, body mass index (BMI), and ethnicity. The patient’s post-operative day zero (POD 0) radiographs of the operated knee were used for measurements. An a priori power analysis was conducted to achieve 80% power at a significance criterion of α = 0.05, which was determined to be 67. Thus, the obtained sample size of *N* = 102 is adequate to test the study hypothesis.

Our primary outcomes of interest were the distances between the array pins and the tibial and femoral implant when the pins were placed intra-incisional, compared to when they were placed extra-incisional.

Distances were obtained by reviewing and measuring using post-operative-day zero radiographs (Antero-Posterior and Lateral views).

**Fig. 1:**
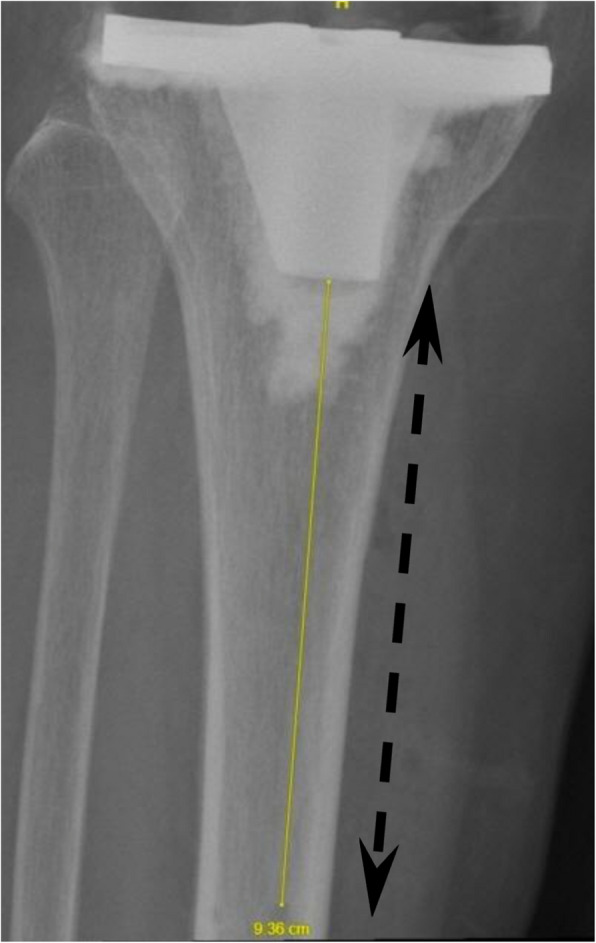
Closest distance from pinhole to tibia tray in anterior–posterior (AP) XR view (measuring 93.6 mm)

**Fig. 2:**
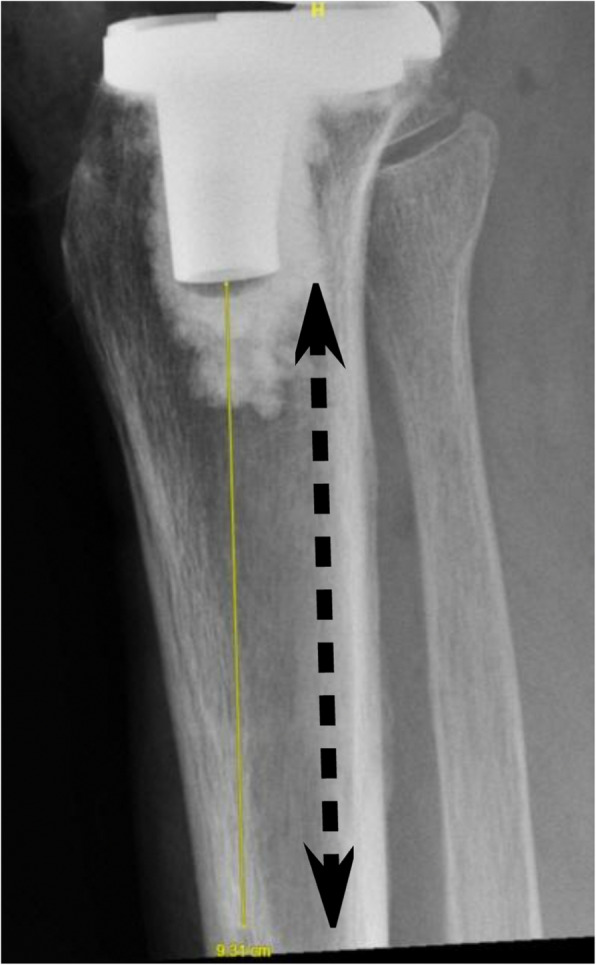
Closest distance from pinhole to tibia tray in lateral view (measuring 93.1 mm)

**Fig. 3:**
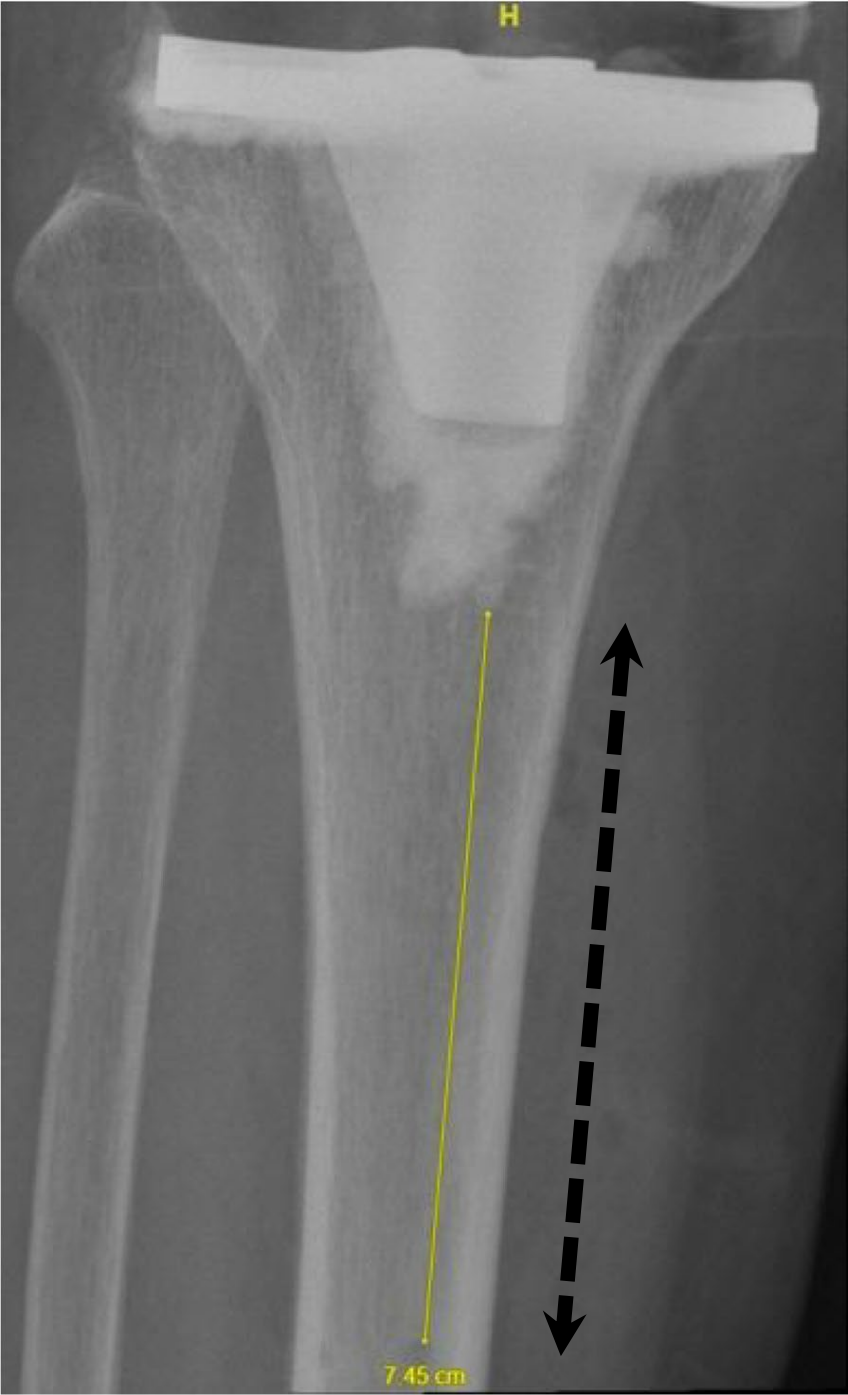
Tibia reamed surface-to-pin distance in AP view (measuring 74.5 mm)

**Fig. 4:**
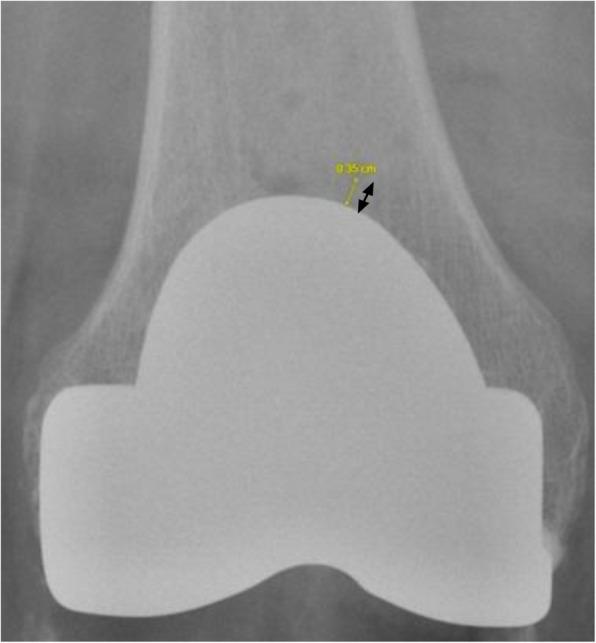
Femur reamed surface-to-pin distance in AP view (measuring 3.5 mm)

Specifically, the distances (mm) measured and defined in the present study are:

- Distance between the closest Tibia Pin and Tibial Implant in AP view (Fig. 1)
- Distance between the closest Tibia Pin and Tibial Implant in Lateral view (Fig. 2)
- Distance between the closest Tibia Pin and the Tibial Reamed Surface in AP (Fig. 3)
- Distance between the closest Femur Pin to the Femoral Reamed Surface (Fig. 4)

To ensure accuracy in measured distances, we accounted for and adjusted distances according to X-ray magnifications.

Secondary outcomes of interest were the complication rates in both groups, such as infections and fractures.

All datasets generated and analysed (including raw data) during the current study are included in this manuscript and its supplementary information files.

### Surgical technique

All 4 pins were placed bi-cortically in the metaphysis of the femur and tibia. The tracker positions were checked to ensure visibility through the range of motion of the knee, including flexion–extension and internal–external rotation as necessary.

#### Femoral pins placement

**Fig. 5:**
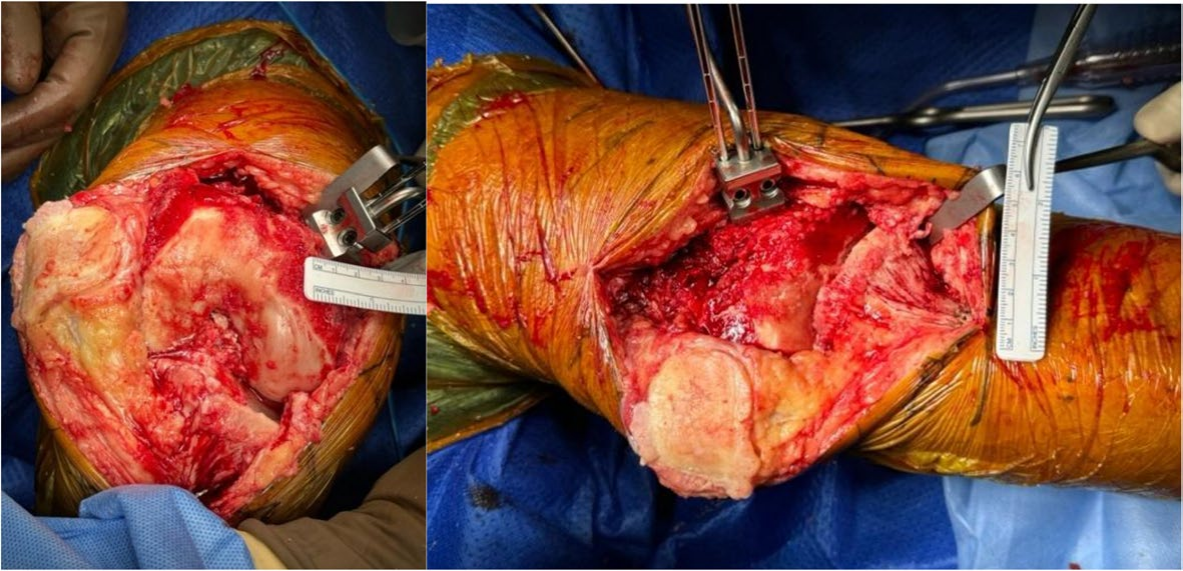
Clinical photo of femoral pins in flexion and extension

The knee is positioned in 90 degrees of flexion (Fig. 5) prior to placement of the femoral pins.

For the placement of the first pin in the distal femur, the starting point is determined by referencing the inflection of the articular border on the medial aspect of the trochlear, before it flares out to form the medial articular condyle, known as the Liau-inflection point. From this reference point, the pin is positioned 20 mm medially and 20 mm proximally. The pin is aimed 10 mm deep to the apex of the intercondylar notch.

The placement of the second pin in the proximal femur is system-specific, as some systems have their arrays arranged longitudinally, while others have them arranged perpendicularly. This second pin is designed to work in conjunction with the first pin, serving the functional purpose of retracting the medial soft tissues to aid in exposure.

The pins are inserted in a trajectory and depth that does not impede the placement of the trial femur component or actual implant. Typically, the pins are placed to engage double cortices for maximal stability. However, during infrequent situations where the pins are over-inserted, which impedes trial implant placement, the pins may be backtracked to just before protruding out of the second cortex and re-tightened.

#### Tibial pins placement

**Fig. 6:**
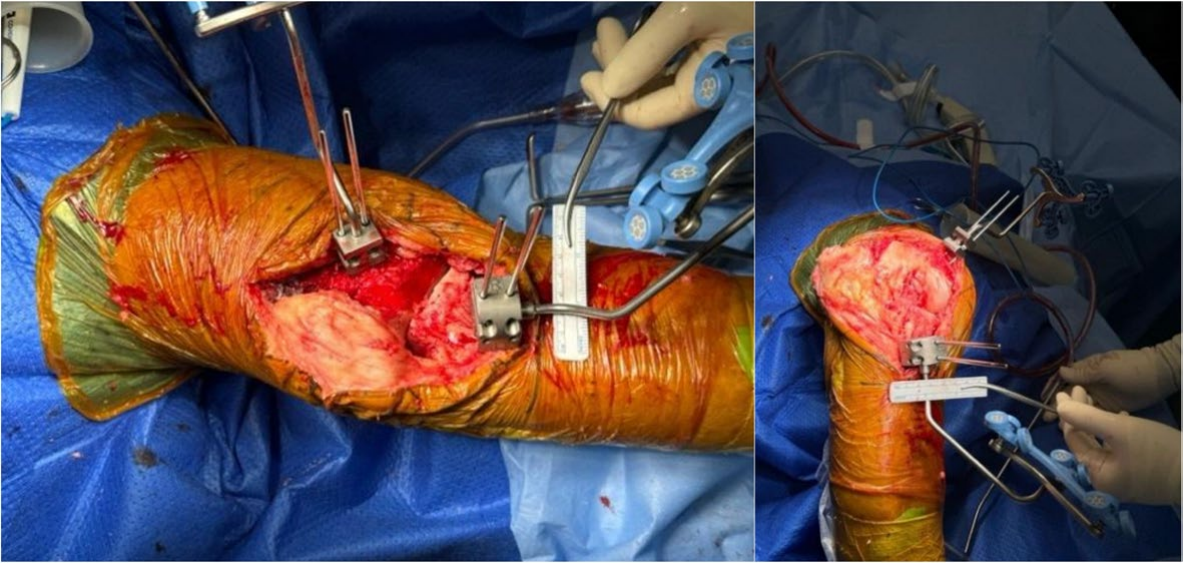
Clinical photo showing tibial and femoral pins in flexion and extension. Distal tibia pins are placed in 15 degrees of medial rotation

The knee is positioned in full extension (Fig. 6) prior to placement of the tibial pins.

For the placement of the first pin in the distal tibia, the assistant retracts the medial soft tissues using two Langenbeck retractors. The distal Langenbeck is positioned to retract the soft tissues distal to the apex of the superficial medial collateral ligament (MCL) without retracting the ligament itself. The proximal Langenbeck retracts the medial soft tissues, including the deep MCL, to ensure proper exposure.

The starting point for the distal tibial pin is determined by referencing the proximal jig hole at the level of the tibial tuberosity and is placed intra-incisionally but extra-capsularly to avoid unnecessary tenting or release of the deep MCL/medial sleeve, which could impact subsequent medial gap balancing assessments.

The distal pin is inserted first, using the distal tracker jig hole, approximately 30 mm from the tibial crest. The pin is aimed 10 degrees distally towards the foot.

For the placement of the second pin in the proximal tibia, the starting point is referenced from the apex of the tibial tuberosity. The pin is placed proximally, about 15 mm away from the tibial tuberosity. The trajectory involves a 10-degree rotation, resulting in an internally rotated tibial tracker jig.

### Statistical analysis

Statistical analyses were performed using RStudio (Version 2022.12.0 + 353).

The Shapiro-Wilks Test was used to determine the normality of the various demographic and outcome data. When data were deemed to be normally distributed, ANOVA and Post-Hoc T-Testing were performed to determine the significance between the desired outcomes. When data were not normally distributed, the Mann–Whitney U, Kruskal–Wallis Tests, and Post-Hoc Dunn Testing were utilised.

Further subgrouping amongst various demographic characteristics (Table 1) was also performed to assess for any possible confounders. We sub-grouped by age (< 70 years old and 70 years or above), Gender (Male/Female), Race, BMI, Implant Type (ROSA Cementless, ROSA Cemented, MAKO, CORI), Height, and Weight.

**Table 1:** Demographic characteristics

	**Age**			**Male %**		**Race (%)**					**Weight**			**Height**			**BMI**			**Implant Type (%)**		
	**Mean (95% CI)**	**Standard Deviation**	***P*-value**	**Mean (95% CI)**	***P*-value**	**Chinese**	**Malay**	**Indian**	**Others**	***P*-value**	**Mean (95% CI)**	**Standard Deviation**	***P*-value**	**Mean (95% CI)**	**Standard Deviation**	***P*-value**	**Mean (95% CI)**	**Standard Deviation**	***P*-value**	**ROSA**	**MAKO**	**CORI**
Intra-incisional (*n* = 53)	69.6 (68, 71.3)	7.6	0.30	40.5%	0.72	66.7	9.5	23.8	0	0.27	71.1 (66.8, 75.3)	13.6	0.63	159 (156, 162)	7.5	0.54	28.1 (26.8, 29.3)	4	0.9	81	11.9	7.1
Extra-incisional (*n* = 49)	68.1 (65.8, 70.5)	6.4		35%		80	3.3	15	1.7		69.7 (65.7, 73.6)	15.2		158 (156, 160)	9		28 (26.5, 29.4)	5.4		40	56.7	3.3

**Fig. 7:**
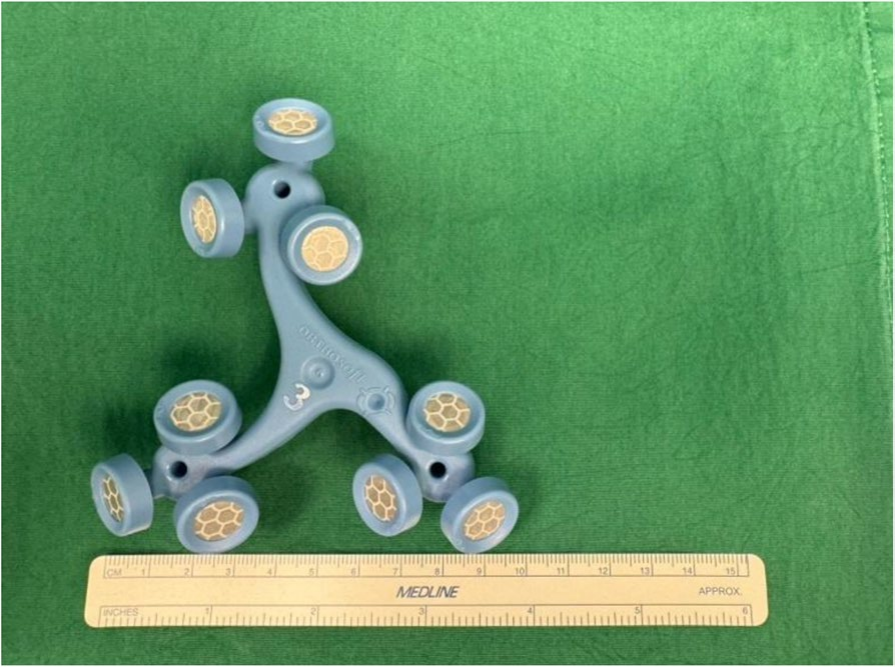
Measurements of the ROSA femur tracker, with surface area 4574 mm^2^* (Note: *calculated using Heron’s formula [16].)

**Fig. 8:**
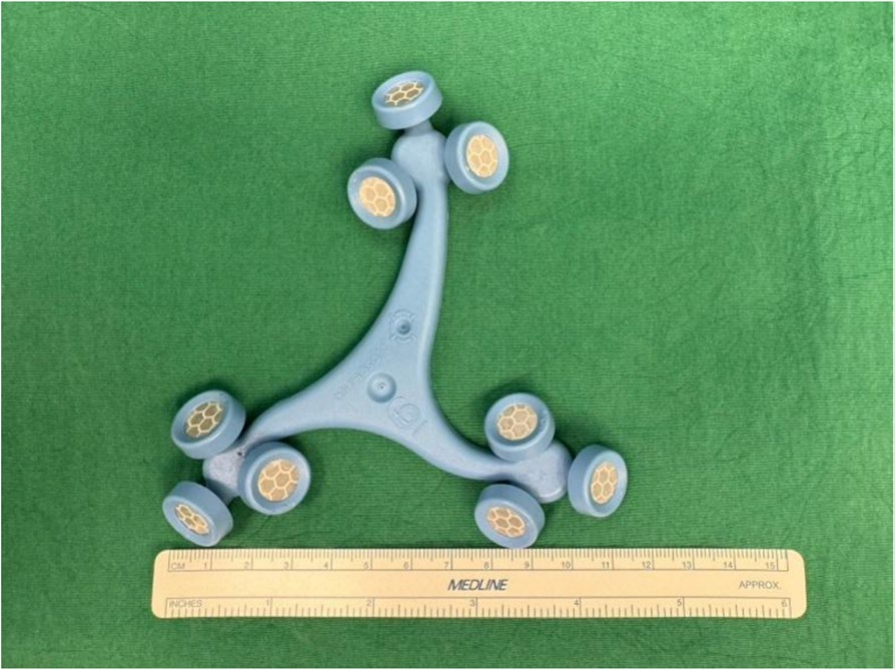
Measurements of the ROSA tibial tracker, with surface area 6358 mm^2^* (Note: *calculated using Heron’s formula [16].)

**Fig. 9:**
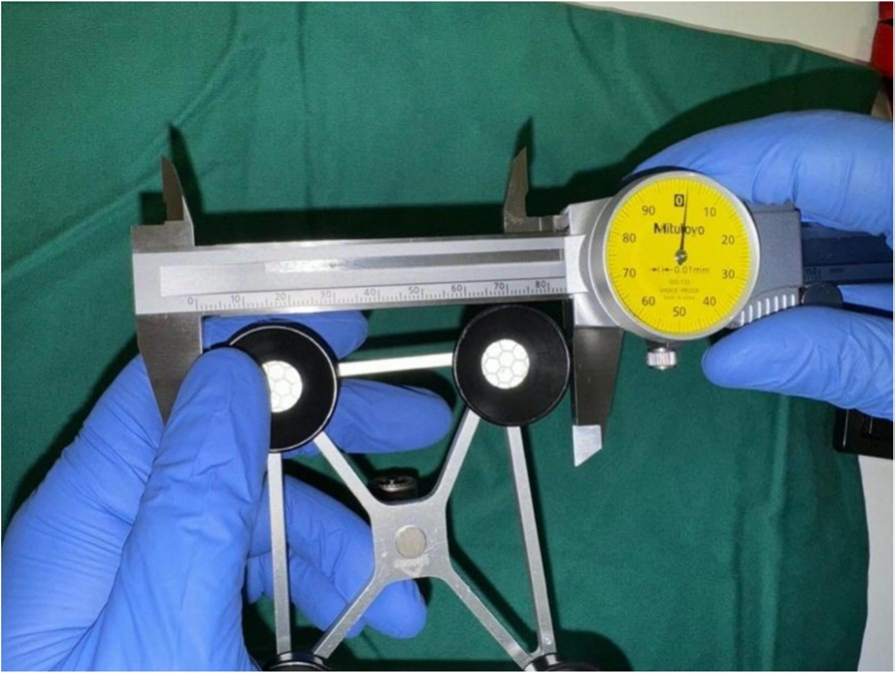
Measurements of the MAKO femur tracker, with surface area 9679 mm^2^** (Note: **calculated using Brahmagupta’s formula [17]., all values rounded off to the nearest whole number)

**Fig. 10:**
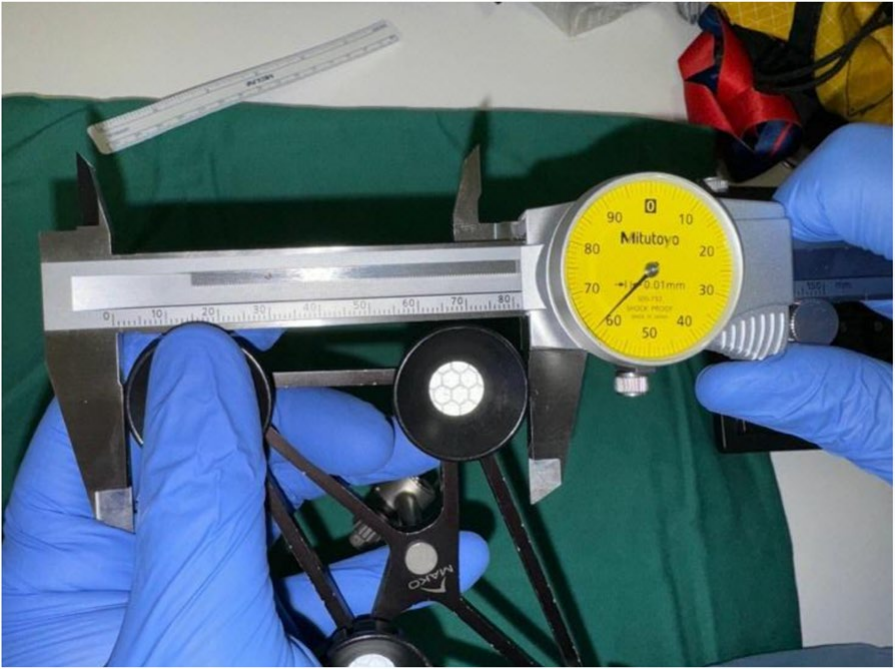
Measurements of the MAKO tibial tracker, with surface area 9607 mm^2^** (Note: **calculated using Brahmagupta’s formula [17]., all values rounded off to the nearest whole number)

**Fig. 11:**
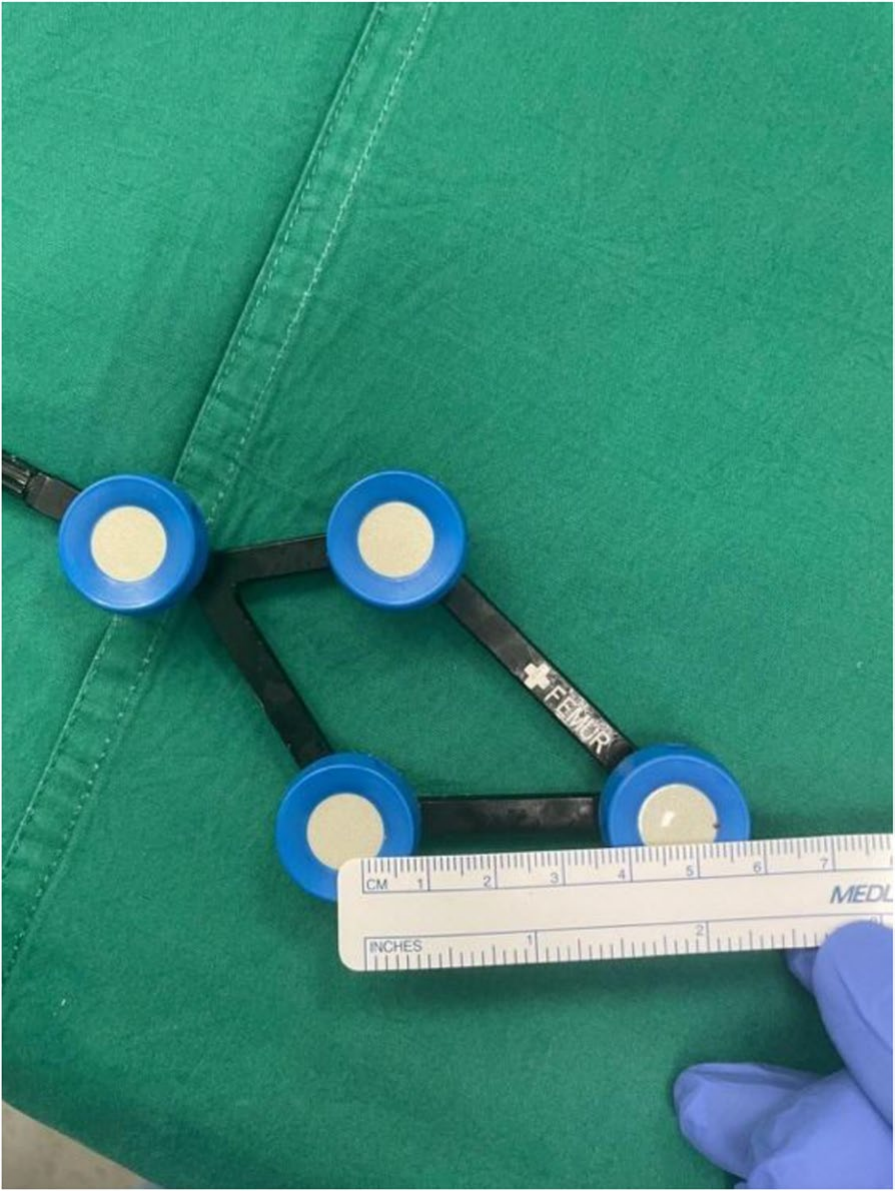
Measurements of the CORI tibia tracker, with surface area 863 mm^2^* (Note: *calculated using Heron’s formula [16].)

**Fig. 12:**
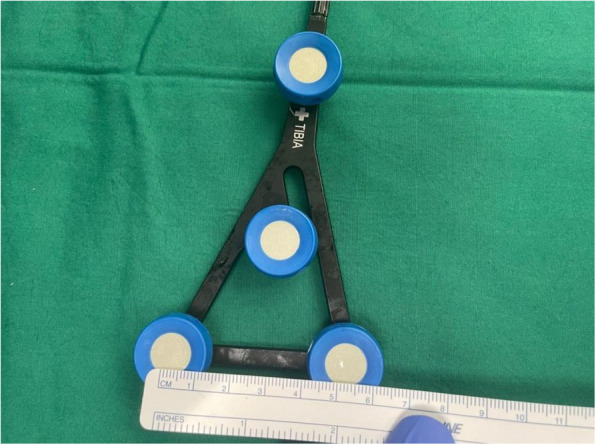
Measurements of the CORI femur tracker, with surface area 2496 mm^2^** (Note: **calculated using Brahmagupta’s formula [17]., all values rounded off to nearest whole number)

Dimensions of the different femoral and tibial trackers for ROSA (Figs. 7 and 8), MAKO (Figs. 9 and 10), and CORI (Figs. 11 and 12) were measured to look for any correlations.

To holistically assess our results, multivariate analysis was also performed to account for age, gender, BMI, race, and robot system.

## Results

A total of 102 robotic-assisted TKAs between February 2023 to May 2024 were included in the analysis of our data.

### Subgrouping

Subgrouping was performed to assess the impact of age, gender, race, BMI, height, weight, and implant types on the parameters of interest.

We found that for the four parameters of interest, Tibia Pin to Tibial Implant on AP View, Tibia Pin to Tibial Implant on Lateral View, Tibia Pin to Tibial Reamed Surface on AP View and Femur Pin to Femoral Reamed Surface on AP View, the aforementioned demographic factors did not significantly impact our primary outcomes, except in three instances:

- In the Extra-incisional group under “Tibial Pin to Tibial Implant in AP View”, the CORI implants resulted in significantly shorter distances compared to the MAKO implants (*P* = 0.03).
- In the extra-incisional pin group under “Femoral Pin to Femoral Reamed Surface in AP View”, the CORI and ROSA implants both resulted in significantly shorter distances than that of the MAKO implant. (*P* = 0.02 and *P* = 0.0008, respectively).
- In the intra-incisional pin group under “Tibial Pin to Tibial Implant in Lateral View”, the ROSA implant resulted in a significantly longer distance than the MAKO implant (*P* = 0.02).

A summary of the remaining subgroups can be found in Table 2

**Table 2:** Subgrouping for age, gender, race, BMI, and implant type

Parameter			Tibial Pin to Tibial Implant on AP View				Tibial Pin to Tibial Implant on Lateral View				Tibial Pin to Tibial Reamed Surface on AP View				Femoral Pin to Femoral Reamed Surface on Lateral View				
**Subgroup**			**Mean Distance (mm)**	**95% CI**	**Standard Deviation**	***P*-Value**	**Mean Distance (mm)**	**95% CI**	**Standard Deviation**	***P*-Value**	**Mean Distance (mm)**	**95% CI**	**Standard Deviation**	***P*-Value**	**Mean Distance (mm)**	**95% CI**	**Standard Deviation**		***P*-Value**
Age	Intra-Incisional Pins	Below 70 years old	9.2	7.28, 11.2	10.2	0.64	10.2	8.24, 12.1	4.3	0.19	6.38	4.43, 8.34	4.1	0.41	5.79	4.06, 7.53		2.1	0.63
		70 years old and Above	8.61	6.65, 10.6	9.6		8.31	6.05, 10.6	4.1		5.35	3.57, 7.13	3.2		6.58	1.03, 12.1		2.2	
	Extra-Incisional Pins	Below 70 years old	55.2	47, 63.3	21.4	0.19	54.8	46.4, 63.2	20.8	0.32	46.7	34.3, 59.2	25	0.92	25.1	15.8, 34.5		21.6	0.31
		70 years old and Above	62.7	54.4, 71.1	19.7		61.1	51.1, 71.1	20.7		47.5	35.8, 59.2	16.3		32.1	21.3, 43		21.8	
Gender	Intra-Incisional Pins	Male	8.44	6.63, 10.3	4.3	0.65	9.19	7.03, 11.4	3.8	0.74	6.18	4.33,8.04	3.4	0.63	6.88	4.66, 9.10		1.5	0.11
		Female	9.8	7.57, 12	4.4		9.67	7.63, 11.7	4.6		5.56	3.61,7.51	4		4.95	3.1, 6.8		2.1	
	Extra-Incisional Pins	Male	32.5	50.3, 66	15.1	0.82	56.1	46.7, 65.4	14.2	0.51	44.2	31.9, 56.4	19	0.38	28.6	16.8, 40.4		16.8	0.86
		Female	59.4	51.6, 67.2	23.2		59.8	52.5, 67.1	24		51.4	38.6,64.2	23.8		27.8	19.9, 35.7		26	
Race	Intra-Incisional Pins	Chinese	9	7.26, 10.8	4.6	0.75	9.9	7.98, 11.8	4.5	0.61	5.94	4.29, 7.59	3.7	0.83	6.02	3.68, 8.36		2.5	0.77
		Indian	10.2	5.07, 16.4	3.2		8	2.22, 13.8	3.6		6.87	1.85, 11.9	3.2		7.38	Only 1 sample			
		Malay	8.29	5.31, 11.3	4.2		8.62	5.53, 11.7	3.7		5.43	1.92, 8.93	4.2		5.52	4.32, 6.71		0.5	
	Extra-Incisional Pins	Chinese	58.9	52.6, 65.3	20.9	0.74	57.5	50.7, 64.2	20.6	0.99	49.9	41.7,58.1	19.4	0.09	28.2	20, 36.5		23.4	0.67
		Indian	66.3	43.9, 88.8	2.5		60.1	Only 1 sample			NA	NA			43.6	−11.2, 98.4		6.1	
		Malay	54.1	32.1, 77.1	23.8		57.1	30.1, 84.1	25.7		29.7	−20.3,79.7	31.4		20.8	5.97, 35.6		11.9	
BMI	Intra-Incisional Pins	Normal	9.1	1.74, 16.5	5.9	0.33	11	8.46, 13.6	1.6	0.64	5.6	−0.03,11.2	4.5	0.47	4.75	1 sample			0.64
		Overweight	8.17	6.46, 18.0	4.3		8.91	7.16, 10.7	4.9		5.37	3.69, 7.05	3.6		4.96	4.31, 7.62		2.4	
		Obese	10.4	7.87, 13	4		9.76	6.04, 13.5	2.5		7.15	4.46, 9.84	4		7.66	1 sample			
	Extra-Incisional Pins	Normal	49.8	32.7, 66.9	22.2	0.22	46.4	29.1, 63.7	20.7	0.13	23.8	7.83,39.8	26	0.30	33.5	9.98, 57		25.4	0.36
		Overweight	63.3	54.1, 72.5	20.7		63.3	53.3, 73.2	21.1		46.3	18.6, 74	17.7		32	21.5, 42.5		21.9	
		Obese	63.3	48.3, 64.9	20.1		55.4	56, 64.9	19.5		49.5	39.7, 59.3	26.6		20.2	9.97, 30.3		20	
Implant	Intra-Incisional Pins	CORI	9.71	2.79, 16.6	2.8	0.84	NA	NA		0.02*	7.99	−3.37, 19.3	4.6	0.50	No Sample				NA
		MAKO	8.11	0.759, 15.5	5.9		5.46	0.96, 9.96	3.6		7.08	−2.06, 16.2	3.7						
		ROSA	9.06	7.75, 10.4	3.8		10	8.57, 11.5	4.0		5.58	4.17,7	3.7		6.01	4.64, 7.37		2.0	
	Extra-Incisional Pins	CORI	24	24, 24.1	0.01	0.03*	24.8	−2.75, 67.5	3.1	0.07	23.8	7.83, 39.8	1.8	0.30	3.59	−7.09, 14.3		1.2	0.0002**
		MAKO	62.6	54.6, 70.6	21		58.3	49, 67.5	21.8		46.3	18.6, 74	26.4		43.2	32.9, 53.5		20.7	
		ROSA	56.6	48.1, 65.1	19.2		59.9	51.3, 68.5	17.8		49.5	39.7, 59.3	20.9		17.7	11.2, 24.2		14.2	

^*^For subgrouping for different implants in the extra-incisional pin group under “Tibial Pin to Tibial Implant in AP View”, *P* = 0.03 indicated significance, so further Tukey Post-Hoc Testing was performed; CORI-MAKO: *P* = 0.03, CORI-ROSA: *P* = 0.08, MAKO-ROSA: *P* = 0.55

^**^For subgrouping for different implants in the extra-incisional pin group under “Femoral Pin to Femoral Reamed Surface in AP View”, *P* = 0.0002 indicated significance, so further Post-Hoc Dunn Testing was performed; CORI-MAKO: *P* = 0.02, CORI-ROSA: *P* = 0.66, MAKO-ROSA: *P* = 0.0008

### Secondary outcomes

We are also keen to look at the rates of complications from both techniques.

## Discussion

**Fig. 13:**
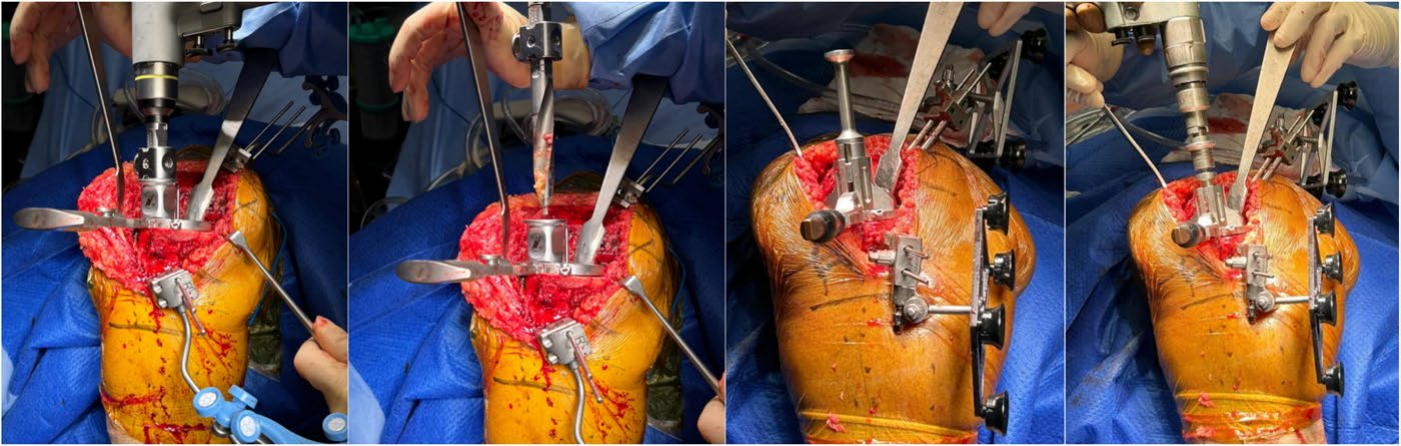
Showing reamer for Zimmer [6] (a and b) and Stryker [5] (c and d) being easily able to pass through for our tibial stem without hitting intra-incisional pins, and with the keel punch completed fully

To the authors’ knowledge, this is the first paper to describe an all-intra-incisional pin technique in the placement of robotic tracker pins in a total knee arthroplasty, regardless of the robotic system used. Our study demonstrates that intra-incisional pins can be placed 6.52 times closer (Table 3) compared to extra-incisional pins, 8.99 mm (95% CI 7.78, 10.2) versus 58.6 mm (95% CI 52.8, 62.4). Employing our techniques, we observed no immediate intra-operative complications and achieved a standard deviation (SD) of 3.9 mm (Table 3, measured on Tibia Pin to Tibial Implant on AP View) for TKAs performed using three different systems (ROSA, CORI, MAKO), as opposed to a SD of 21.1 mm using extra-incisional pins. Additionally, there was no need to reposition (Fig. 13) any pins during the procedures when utilizing intra-incisional pins.

**Table 3:** Results and multivariate analysis

Parameter		Mean distance (mm)	95% CI	Standard deviation	*P*-Value	Multivariate analysis
						**Adjusted *P*-Value**
Tibia Pin to Tibial Implant on AP View	Intra-incisional pins	8.99	7.78, 10.2	3.9	** < 0.001**	** < 0.001**
	Extra-incisional pins	58.6	52.8, 64.4	21.1		
Tibia Pin to Tibial Implant on Lateral View	Intra-incisional pins	9.40	7.97, 10.8	4.2	** < 0.001**	** < 0.001**
	Extra-incisional pins	57.5	51.2, 63.7	20.8		
Tibia Pin to Tibial Reamed Surface on AP View	Intra-incisional pins	5.93	4.64, 7.22	3.7	** < 0.001**	** < 0.001**
	Extra-incisional pins	47.0	38.5, 55.5	22		
Femur Pin to Femoral Reamed Surface on AP View	Intra-incisional pins	6.01	4.64, 7.37	2	**0.002**	** < 0.001**
	Extra-incisional pins	28.2	21.3, 35.1	21.7

The lateral tibial pin-to-implant distance in the MAKO system was significantly shorter than in ROSA and CORI, despite MAKO having a larger tracker surface area (Tables 4 and 5). This indicates that our surgical technique is not influenced by the size of the tracker surface. Similarly, as shown in Table 6, even when comparing the total tracker surface area across the robotic systems, the tibial pin-to-implant distance remained smallest for MAKO. While there was limited data available for the MAKO and CORI systems to do a meaningful statistical analysis, our findings reinforce that the accuracy of our technique is independent of tracker surface dimensions.

**Table 4:** Showing the relationship between the surface area of femoral trackers of the different systems and the distances measured

Pin Placement	Implant	Surface Area	Tibial Pin to Tibial Implant on AP View				Tibial Pin to Tibial Implant on Lateral View				Tibial Pin to Tibial Reamed Surface on AP View				Femoral Pin to Femoral Reamed Surface on Lateral View					
			**Mean**	**95% CI**	**Standard Deviation**	***P*-Value**	**Mean**	**95%CI**	**Standard Deviation**	***P*-Value**	**Mean**	**95% CI**	**Standard Deviation**	***P*-Value**	**Mean**	**95% CI**		**Standard Deviation**		***P*-Value**
Intra-Incisional	ROSA	24.2 cm^2^	9.06	7.75, 10.4	3.8	**0.4**	10	8.57, 11.5	4.0	**0.02**	5.58	4.17,7	3.7	0.8	6.01		4.64, 7.37		2.0	NA
	MAKO	43.6cm^2^	8.11	0.759, 15.5	5.9		5.46	0.96, 9.96	3.6		7.08	−2.06, 16.2	3.7		NA		NA		NA	
	CORI	18.8cm^2^	9.71	2.79, 16.6	2.8		NA	NA	NA		7.99	−3.37, 19.3	4.6		NA		NA		NA	
Extra-Incisional	ROSA	24.2cm^2^	56.6	48.1, 65.1	19.2	0.06	59.9	51.3, 68.5	17.8	0.6	49.5	39.7, 59.3	20.9	0.9	17.7		11.2, 24.2		14.2	**0.01**
	MAKO	43.6cm^2^	62.6	54.6, 70.6	21		58.3	49, 67.5	21.8		46.3	18.6, 74	26.4		43.2		32.9, 53.5		20.7	
	CORI	18.8cm^2^	24	24, 24.1	0.01		24.8	−2.75, 67.5	3.1		23.8	7.83, 39.8	1.8		3.59		−7.09, 14.3		1.2

**Table 5:** Showing the relationship between the surface area of tibia trackers of the different systems and the distances measured

Pin Placement	Implant	Surface Area	Tibial Pin to Tibial Implant on AP View				Tibial Pin to Tibial Implant on Lateral View				Tibial Pin to Tibial Reamed Surface on AP View				Femoral Pin to Femoral Reamed Surface on Lateral View						
			**Mean**	**95% CI**	**Standard Deviation**	***P*-Value**	**Mean**	**95%CI**	**Standard Deviation**	***P*-Value**	**Mean**	**95% CI**	**Standard Deviation**	***P*-Value**	**Mean**	**95% CI**		**Standard Deviation**		***P*-Value**	
Intra-Incisional	ROSA	31.8cm^2^	9.06	7.75, 10.4	3.8	0.5	10	8.57, 11.5	4.0	**0.02**	5.58	4.17,7	3.7	0.7	6.01		4.64, 7.37		2.0		NA
	MAKO	49.4cm^2^	8.11	0.759, 15.5	5.9		5.46	0.96, 9.96	3.6		7.08	−2.06, 16.2	3.7		NA		NA		NA		
	CORI	8.6cm^2^	9.71	2.79, 16.6	2.8		NA	NA	NA		7.99	−3.37, 19.3	4.6		NA		NA		NA		
Extra-Incisional	ROSA	31.8cm^2^	56.6	48.1, 65.1	19.2	**0.02**	59.9	51.3, 68.5	17.8	0.2	49.5	39.7, 59.3	20.9	0.4	17.7		11.2, 24.2		14.2	**0.01**	
	MAKO	49.4cm^2^	62.6	54.6, 70.6	21		58.3	49, 67.5	21.8		46.3	18.6, 74	26.4		43.2		32.9, 53.5		20.7		
	CORI	8.6cm^2^	24	24, 24.1	0.01		24.8	−2.75, 67.5	3.1		23.8	7.83, 39.8	1.8		3.59		−7.09, 14.3		1.2

**Table 6:** Showing the relationship between the combined surface areas of femoral and tibia trackers of the different systems and the distances measured

Pin Placement	Implant	Surface Area	Tibial Pin to Tibial Implant on AP View				Tibial Pin to Tibial Implant on Lateral View				Tibial Pin to Tibial Reamed Surface on AP View				Femoral Pin to Femoral Reamed Surface on Lateral View			
			**Mean**	**95% CI**	**Standard Deviation**	***P*-Value**	**Mean**	**95%CI**	**Standard Deviation**	***P*-Value**	**Mean**	**95% CI**	**Standard Deviation**	***P*-Value**	**Mean**	**95% CI**	**Standard Deviation**	***P*-Value**
Intra-Incisional	ROSA	56cm^2^	9.06	7.75, 10.4	3.8	0.5	10	8.57, 11.5	4.0	**0.02**	5.58	4.17,7	3.7	0.9	6.01	4.64, 7.37	2.0	NA
	MAKO	93cm^2^	8.11	0.759, 15.5	5.9		5.46	0.96, 9.96	3.6		7.08	−2.06, 16.2	3.7		NA	NA	NA	
	CORI	27.4cm^2^	9.71	2.79, 16.6	2.8		NA	NA	NA		7.99	−3.37, 19.3	4.6		NA	NA	NA	
Extra-Incisional	ROSA	56cm^2^	56.6	48.1, 65.1	19.2	0.2	59.9	51.3, 68.5	17.8	0.4	49.5	39.7, 59.3	20.9	0.6	17.7	11.2, 24.2	14.2	**0.01**
	MAKO	93cm^2^	62.6	54.6, 70.6	21		58.3	49, 67.5	21.8		46.3	18.6, 74	26.4		43.2	32.9, 53.5	20.7	
	CORI	27.4cm^2^	24	24, 24.1	0.01		24.8	−2.75, 67.5	3.1		23.8	7.83, 39.8	1.8		3.59	−7.09, 14.3	1.2	

In fact, in the group with extra-incisional pins, there was one instance of a potential pin-site related tibia fracture (2.04%), whereas there was none for the intra-incisional group (Table 7), suggesting intra-incisional pins might reduce pin-related complications.

**Table 7:** Comparing rates of complications between the intra-incisional group and the extra-incisional group

	**Intra-incisional, *n* (%)**	**Extra-incisional, *n* (%)**
Complications * (including infections and peri-prosthetic fractures)	0	1 (2.04)

^*^ Fracture involving the tibia propagating from the tibia pin site

It has been shown that pins in the diaphysis are more likely to cause stress risers, which may lead to pin site fractures [9, 10]. This is hypothesized by diaphysis having a smaller surface area and pins passing through non-cancellous bone. Our technique allows pins to go through the metaphyseal region of both femur and tibia, which have a larger surface area and cancellous bone. Metaphyseal bone is also more robust and can withstand a higher degree of rotational stress [18], with Hyun et al. modifying their approach in their institution for pin placements in the metaphysis. This reduces the potential for stress risers and consequently the risk of pin-site fractures [9, 10]. In the authors’ opinion, this is a safer technique to employ when inserting robotic TKA tracker pins.

There were limitations to our study. Firstly, there is an associated learning curve with the described technique. While the exact number of cases required to achieve proficiency has not been formally evaluated, it is anticipated to be minimal. Based on the author's anecdotal experience, competency was attained after fewer than 10 cases, suggesting the technique is relatively straightforward to adopt.

Secondly, the intra-incisional femur pin holes were mostly not visible on the lateral X-rays. Consequently, we were unable to assess the pin-to-femur implant distances. This can be due to multiple factors, including the size of the metaphysis, the trajectory angle of the femur pins, and the visualization of the far cortical purchase, which could be obstructed by the condyles of the femur implant.

Another limitation is that the X-ray images were captured at varying distances from the operated knee and from different angles. This inconsistency can lead to varied projections of X-ray beams on the knee, resulting in measurements that may not accurately reflect the true distances. Although we mitigated this issue by employing a standard magnification, the results might still lack the precision of computed tomography (CT) images, which track the entire trajectory. However, due to concerns about radiation exposure and financial constraints, we did not perform post-operative CT scans.

Lastly, the all-intra-incisional-pins technique was performed by only a single surgeon, and the reproducibility of this technique by other surgeons needs to be evaluated. Additionally, we were unable to incorporate other robotic TKA systems, such as VELYS [19, 20], into our study as they were not available at our institution during the study period.

## Conclusion

Our study has demonstrated that our technique is consistent and easily reproducible in achieving precision and is compatible with all major robotic TKA systems. This technique has resulted in a reduced incidence of pin-site fracture complications. This technique offers a viable and safer alternative for robotic TKAs.

## Supplementary Information

Supplementary Material 1.

## Data Availability

All data will be made available upon reasonable request.
