# Supplementary material for: Novel all-intra-incisional pin placement technique in robotic total knee arthroplasty: a safer alternative
Source: Arthroplasty. 2025 Sep 2;7:45. doi: 10.1186/s42836-025-00329-8 (PMC12403252; doi:10.1186/s42836-025-00329-8)
Supplement: Supplementary file 1 — Supplementary Material 1. [file 42836_2025_329_MOESM1_ESM.docx]

| **Parameter** | | | **Tibial Pin to Tibial Implant on AP View** | | | | **Tibial Pin to Tibial Implant on Lateral View** | | | | **Tibial Pin to Tibial Reamed Surface on AP View** | | | | **Femoral Pin to Femoral Reamed Surface on Lateral View** | | | | | |
| --- | --- | --- | --- | --- | --- | --- | --- | --- | --- | --- | --- | --- | --- | --- | --- | --- | --- | --- | --- | --- |
| Subgroup | | | Mean Distance (mm) | 95% CI | Standard Deviation | P-Value | Mean Distance (mm) | 95% CI | Standard Deviation | P-Value | Mean Distance (mm) | 95% CI | Standard Deviation | P-Value | Mean Distance (mm) | 95% CI | Standard Deviation | | P-Value |  |
| Height | Intra-Incisional Pins | Below 160cm | 8.50 | 6.96, 10 | 4 | 0.31 | 8.83 | 6.88, 10.8 | 4.5 | 0.34 | 6.05 | 3.9, 8.2 | 4 | 0.89 | 6.75 | 4.73, 8.77 | | 2.2 | 0.06 | |
|  |  | 160cm and Above | 9.8 | 7.64, 12 | 3.7 |  | 10.2 | 7.91, 12.5 | 3.9 |  | 5.86 | 4.12, 7.6 | 3.4 |  | 4.7 | 3.39, 6.01 | | 0.8 |  |  |
|  | Extra-Incisional Pins | Below 160cm | 59.4 | 51.4, 67.4 | 21.6 | 0.62 | 56.4 | 47, 65.8 | 24.7 | 0.61 | 53.4 | 40.7, 66 | 23.9 | 0.23 | 28.9 | 18.9, 39 | | 24.3 | 0.90 | |
|  |  | 160cm and Above | 56.9 | 50.1, 63.6 | 11.8 |  | 59.3 | 52.3, 66.3 | 13.3 |  | 43.5 | 31.7, 55.3 | 17 |  | 27.1 | 17.7, 36.5 | | 17.7 |  |  |
| Weight | Intra-Incisional Pins | Below 70kg | 9.42 | 7.35, 11.5 | 4.3 | 0.63 | 9.25 | 6.59, 11.9 | 4.8 | 0.86 | 6.51 | 4.24, 8.78 | 4.3 | 0.41 | 5.31 | 3.4, 7.22 | | 1.5 | 0.31 | |
|  |  | 70kg and Above | 8.64 | 7.08, 10.2 | 3.6 |  | 9.52 | 7.74, 11.3 | 3.9 |  | 5.41 | 3.85, 6.97 | 3.1 |  | 6.58 | 4.12, 9.05 | | 2.4 |  |  |
|  | Extra-Incisional Pins | Below 70kg | 59 | 51.2, 66.7 | 22.2 | 0.87 | 55.5 | 46.5, 64.4 | 22.7 | 0.41 | 49.9 | 40.4, 59.5 | 19.2 | 0.41 | 28.8 | 18.8, 38.8 | | 24.2 | 0.84 | |
|  |  | 70kg and  Above | 58 | 48.7, 67.3 | 19.4 |  | 60.5 | 51.7, 69.3 | 17.7 |  | 41.8 | 40.4, 59.5 | 26.5 |  | ~~27.3~~ | 17.8, 36.9 | | 17.9 |  |  |

Table 6: Sub-group analysis of height and weight
